# HBV-Associated Postinfectious Acute Glomerulonephritis: A Report of 10 Cases

**DOI:** 10.1371/journal.pone.0160626

**Published:** 2016-08-11

**Authors:** Yong Zhang, Junxia Li, Weihua Peng, Guoqing Yu, Liping Wang, Jian Chen, Feng Zheng

**Affiliations:** 1 Department of Nephrology and Medicine, Dongfang Hospital, Fujian Medical University, Fuzhou, Fujian, China; 2 Department of Nephrology, The Second Hospital, and Advanced Institute for Medical Sciences, Dalian Medical University, Dalian, China; Hopital universitaire Necker-Enfants malades, FRANCE

## Abstract

Postinfectious acute glomerulonephritis (PIGN) may occur after various bacterial and viral infections. Hepatitis B virus (HBV) infection is a cause of chronic glomerulonephritis. We report here 10 cases (ages 7–20 years-old) of chronic HBV carriers with acute glomerulonephritis, with positive glomerular staining of hepatitis B surface antigen, and detectable presence of HBV DNA in the glomeruli. This form of PIGN, HBV-PIGN, has not been previously identified. To further characterize clinical and pathological features of HBV- PIGN, we selected 10 cases of age-matched non-HBV PIGN for comparison. While both HBV associated PIGN and non-HBV PIGN similarly presented as proteinuria, hematuria, and hypertension, there was a trend of higher acute kidney injury and worsened prognosis in HBV-PIGN. 6 months after the onset, 4 patients with HBV associated PIGN did not show improvement from the disease, whereas all patients with non-HBV PIGN had complete or partial recovery. Pathologically, both HBV associated PIGN and non-HBV PIGN showed typical diffuse glomerular endocapillary proliferation, but HBV associated PIGN differed from classical PIGN with much fewer sub-epithelial glomerular “hump-shape” immune complex depositions. In conclusion, we have identified a novel association of HBV infection with acute glomerulonephritis.

## Introduction

Post-infectious glomerulonephritis (PIGN) is an immune-mediated glomerular disease that predominantly occurs in children following infection in the upper respiratory or skin [1,2]. Recently however, increasing prevalence of PIGN has also been found in immunocompromised adults and other older individuals, particularly in conjunction with diabetes and other morbidities [3, 4]. While it is difficult to estimate the precise prevalence of PIGN due to underreporting, PIGN remains a problem in the developing world ^(1)^. The underlying mechanisms of PIGN are obscure. Several different types of pathogens have been linked to PIGN, with bacteria such as streptococcus and staphylococcus being especially common instigators [1]. Various viruses such as human immunodeficiency virus, Epstein-Barr virus, hepatitis C virus, and human parvovirus B19 have also been implicated in PIGN [5, 6]. PIGN may be especially common in tropical areas that are more advantageous for transmission [2].

Hepatitis B virus (HBV) is a member of the family hepadnaviridae that is endemic in many areas across the world. HBV causes transient and/or persistent infection in the liver after its incorporation, relying on hepatocytes to replicate itself. HBV may also infect extrahepatic tissue such as the bile duct, the pancreas, the lymphoid system, and the kidneys [7]. It is well recognized that HBV infection may cause chronic glomerulonephritis, although the underlying molecular mechanism of the pathogenesis, including the exact contributions of HBV, remains largely unknown [8–11]. The clinical manifestation of HBV associated chronic glomerulonephritis (HBV-GN) has been difficult to distinguish from other forms of glomerulonephritis, and might present as nephritic syndrome, mild to moderate proteinuria, hematuria, or renal function insufficiency. Membranous nephropathy is the most common pathologic type of HBV-GN. HBV has also been linked to other glomerular diseases such as IgA nephropathy, membranous proliferative glomerulonephritis, and mesangial proliferative glomerulonephritis _[__8__–__11__]._

In this report, we present for the first time 10 cases of HBV associated acute PIGN. Through clinical manifestations, laboratory findings, renal pathology and clinical outcome after 6 months of diagnosis of the disease, we report some significant differences between HBV associated acute PIGN and classical acute PIGN. In particular, we identify some differences in the immune complex that could underlie the overall contrast in disease severity between HBV associated acute PIGN and classical acute PIGN.

## Patients and Methods

From January 2005 to December 2013, 10 cases (7–20 years old, mean age 13 years old) were diagnosed with HBV associated acute endocapillary proliferative glomerulonephritis (HBV-PIGN) in our pathology center. The diagnosis criteria for HBV-PIGN were: 1) typical pathology of diffuse glomerular endocapillary proliferation, including marked hypercellularity with visible neutrophil infiltration under light microscopy and the appearance of “hump-shaped” sub-epithelial, electron-dense deposits under electron microscopy, 2) positive test for serum hepatitis B surface antigen (HBsAg), 3) positive glomerular staining for HBsAg with or without hepatitis B core antigen (HBcAg), 4) no history of primary or secondary kidney disease. We have also selected for comparison as control 10 age-matched cases (5–24 years old, mean age 12 years old) from the same time period. These age-matched cases had acute PIGN with negative glomerular staining for HbsAg and negative for serum HBsAg. This study was approved by the Institutional Review Board (IRB) of Dongfang Hospital. Renal biopsies and the use of biopsy tissues had the approval of patients or their legal representatives with written informed consent.

Clinical information including demographic data, medical history, clinical presentation and laboratory results were collected. Specifically, serology tests for HBV, deoxyribonucleic acid (DNA) of HBV, hepatitis C, human immunodeficiency virus, and antistreptolysin O antibody were performed. Urine and orolaryngeal swab culture were also performed. The following clinical classifications were used: Acute nephritic syndrome—acute onset hematuria and proteinuria (<3 g/day); hypertension—systolic blood pressure>140 mmHg and diastolic blood pressure>90 mmHg; nephrotic syndrome—proteinuria>3 g/day, hypoalbuminemia—(serum albumin <2.5 g/dL), edema, and hyperlipidemia; Acute kidney injury—rapidly increase in serum creatinine≥26.5 μmol within 48 hours.

Antibiotics were given to patients with non-HBV associated PIGN who tested positive for oropharyngeal swab bacterial culture or for antistreptolysin O. In patients with HBV-PIGN, lamivudine was given to serum HBeAg positive patients and/or patients with circulating HBV DNA titer higher than 2000 [12,13]. Diuresis, antihypertensive, anticoagulation, and other symptom-based treatments were also given to patients with both HBV-PIGN and non-HBV associated PIGN. Additionally, three patients with nephrotic syndrome, one in HBV-PIGN and two in non-HBV associated PIGN were given glucocorticoid. Hemodialysis was initiated in patients with severe acute kidney injury (serum creatinine levels greater than 618 μmol).

### Renal biopsy and Pathology

Renal biopsy was performed at an average of 7 days (3–14 days) in HBV-PIGN and of 10 days (4–19 days) in non-HBV associated acute PIGN, respectively, after the onset of renal disease. Renal tissues were processed for light microscopy, immunoperoxidase staining, and electron microscopy using standard protocols in our center as described previously [14]. Immunostaining for IgG, IgM, IgA, C3, C4, C1q, fibrinogen, HBsAg, and HBcAg was routinely performed with EliVision two step immunoperoxidase kit. Primary peroxidase-conjugated polyclonal antibodies against the respective targets were purchased from DAKO Corp, Denmark.

### Glomerular HBV DNA

Glomeruli were microdissected from renal biopsy under a dissecting microscope as previously described [15]. Two glomeruli from each patient were collected in a PCR tube with 10 μl of DNA isolation solution containing 0.1% Triton-x100 and 200 μg/ml of proteinase K and were incubated at 55°C for 3 hours. After inactivating proteinase K at 95°C for 10 minutes, supernatant was collected by centrifugation at 13000 rpm for 5 minutes. To detect HBV DNA in glomeruli, 4 μl of supernatant was added to a 25 μl PCR reaction that included dNTP, Taq DNA polymerase, MgCl_2_, and 10×buffer, and HBV DNA specific primers (Forward, 5’-TTCAAGCCTCCAAGCTGTGCCTTGGA; Reverse, 5’-CACCCAGGTAGCTAGAGTCATTAA). The specificity of HBV DNA PCR was confirmed by sequencing. Briefly, the 253 bp PCR product was cut from agarose gel. QIAquick gel extraction kit was used to obtain purified PCR product for automated sequencing. PCR for β-actin was performed at the same time as control.

### Statistical analysis

To examine the differences between HBV-PIGN and non HBV associated PIGN, patients’ characteristics including baseline demographics, clinical manifestations, and renal pathology were evaluated by χ^2^ test. Quantitative data was expressed as mean±SD.

## Results

### 1. Clinical features and laboratory findings

The mean age was 13 years old in patients with HBV-PIGN, consistent with the fact that PIGN occurs more commonly in children. The mean age was 12 years old in non-HBV associated PIGN (Table 1). There were 8 males and 2 females in the HBV-PIGN group and 4 males and 6 females in non-HBV associated PIGN group. All10 patients with HBV-PIGN were positive for serum HBsAg and HBeAg and HBcAb at the onset of acute nephritis. Four had high titer of circulating HBV DNA ranging from 4 to 9.5×10^6^ copies/ml. However, only one patient had a mild liver function abnormality, with a small increase in serum aspartate aminotransferase and alanine aminotransferase activities. It was noted that three patients with HBV-PIGN had skin impetigo before PIGN, although the causative bacteria was not identified. None of 10 HBV-PIGN patients had current, or a history of, acute hepatitis B infection. Serological HBV infection markers were negative at birth. In addition, there were no signs of acute flare of chronic hepatitis B, such as jaundice, increased bilirubin, continual and gradual elevation of liver enzyme activity, or severe thrombocytopenia.

**Table 1:** General charactersitics of patients with HBV and non-HBV associated PIGN.

	HBV-PIGN (n = 10)	non-HBV PIGN (n = 10)
Age (years)	13±5	12±7
Sex (M/F)	8/2	6/4
Clinical presentation (case)		
Nephritic syndrome	9	8
Nephrotic syndrome	1	2
Gross hematuria	4	6
Hypertension	3	6
Acute kidney injury	4	2
Blood tests		
High ASO (titer)	167.3±29.5	488.2±140.4
HBsAg/HBeAg/DNA (case)	10/10/4	0/0/0
Low C3/C4 (case)	10/5	10/10
High/low IgG (case)	4/0	0/2
Serum creatinine (μM)	226.4±222.5	216.9±306.8
Serum albumin (g/L)	28.59±5.94	28.81±8.18

The definition of clinical presentation was described in the text. Abbreviation: ASO, anti-streptolysin O; C3, complement 3; C4, complement 4.

By contrast, only one patient of the 10 with non-HBV associated PIGN had positive serum HBcAb, and that patient had undetectable circulating HBV DNA. Importantly, nine patients with non-HBV associated PIGN had signs of acute upper respiratory infection, including cough, sore throat, nasal congestion, and fever, before the onset of kidney disease. Serum anti-streptolysin O levels were elevated in 9 patients with non-HBV associated PIGN, and were normal in all HBV-PIGN patients. Streptococcus was identified to be the causative agent for acute upper respiratory infection in five out of nine patients with non-HBV associated PIGN.

Acute nephritic syndrome, characterized by acute onset hematuria and proteinuria, was the most common manifestation in both HBV-PIGN (9 patients) and non-HBV associated PIGN (8 patients) (Table 1). Acute kidney injury was observed in 4 patients with HBV-PIGN (serum creatinine 136–646 μmol) and 2 patients with non-HBV associated PIGN (serum creatinine 503–1004 μmol). Nephrotic syndrome was present in one patient with HBV-PIGN and two patients with non-HBV associated PIGN and was treated with glucocorticoid. 4 patients with HBV-PIGN and 6 patients with non-HBV associated PIGN had macroscopic hematuria. Hypertension occurred in 3 patients with HBV-PIGN and 6 patients with non-HBV associated PIGN. Overall then, it seems that the clinical manifestations of HBV-PIGN and non-HBV associated PIGN are largely similar, which is not altogether unexpected.

Interestingly however, while the levels of complement 4 were lower in all cases of non-HBV associated acute PIGN, they were normal in 5 patients with HBV-PIGN. Serum immunoglobulin G levels were also elevated in 4 patients with HBV-PIGN, whereas the levels were reduced in 2 patients with non-HBV associated PIGN. The levels of complement 3 decreased in both HBV-PIGN and non-HBV associated PIGN. The cause(s) of these differences between HBV-PIGN and non-HBV associated PIGN are not clear.

### 2. Renal pathology

**Fig 1:**
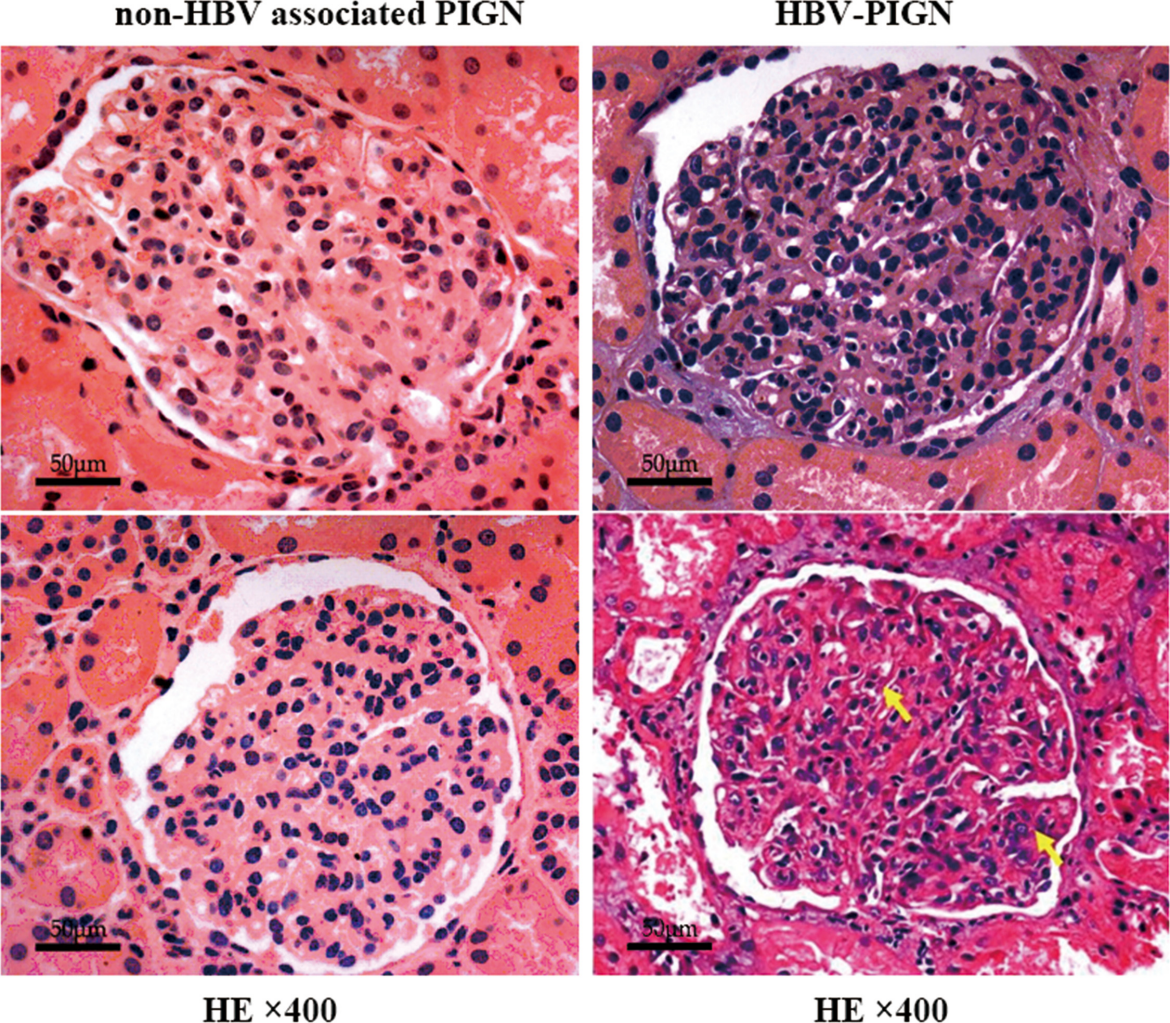
Diffuse endocapillary proliferation in HBV-PIGN and non-HBV associated PIGN: Representative glomerular pathology shows increased cell counts in both the glomerular capillary and mesangium (arrows). The opening of glomerular capillary is noticeably reduced in breadth. Glomerular pathology was indistinguishable between non-HBV associated PIGN (A, B) and HBV-PIGN (C, D).

Diffuse glomerular endocapillary proliferation, a pathologic feature of acute post-infectious glomerulonephritis, was present in all 10 cases of HBV-PIGN (Fig 1). Glomerular size was enlarged due to increased cellularity. There was noticeable neutrophil infilatration inside glomerular capillary, together with glomerular endothelial cell proliferation, resulting in the occlusion of some of the capillary loops. Mild to moderate mesangial proliferation was observed in 5 cases with HBV-PIGN, while the other 5 cases were without obvious mesangial lesions. Cellular crescents were found in a patient with HBV-PIGN, occurring in 12% of glomeruli (3 out of 25 glomeruli). Glomerular basement membranes were largely normal with respect to shape and thickness. Bowman’s capsules were mostly intact, with normal cell counts and appearance. Swollen tubular cells and interstitial edema were observed in 6 out of 10 patients with HBV-GN, and 4 out of 10 patients with non-HBV associated PIGN. Secondary acute kidney injury was present in 4 patients with HBV-PIGN and 2 patients with non-HBV associated PIGN. Interstitial inflammatory lesions characterized by the infiltration of inflammatory cells (including neutrophils, lymphocytes and monocytes) were occasionally seen in patients with HBV-GN and non-HBV associated PIGN, although the lesions were mild overall. Vessels in the tubulointerstitium were largely normal.

**Fig 2:**
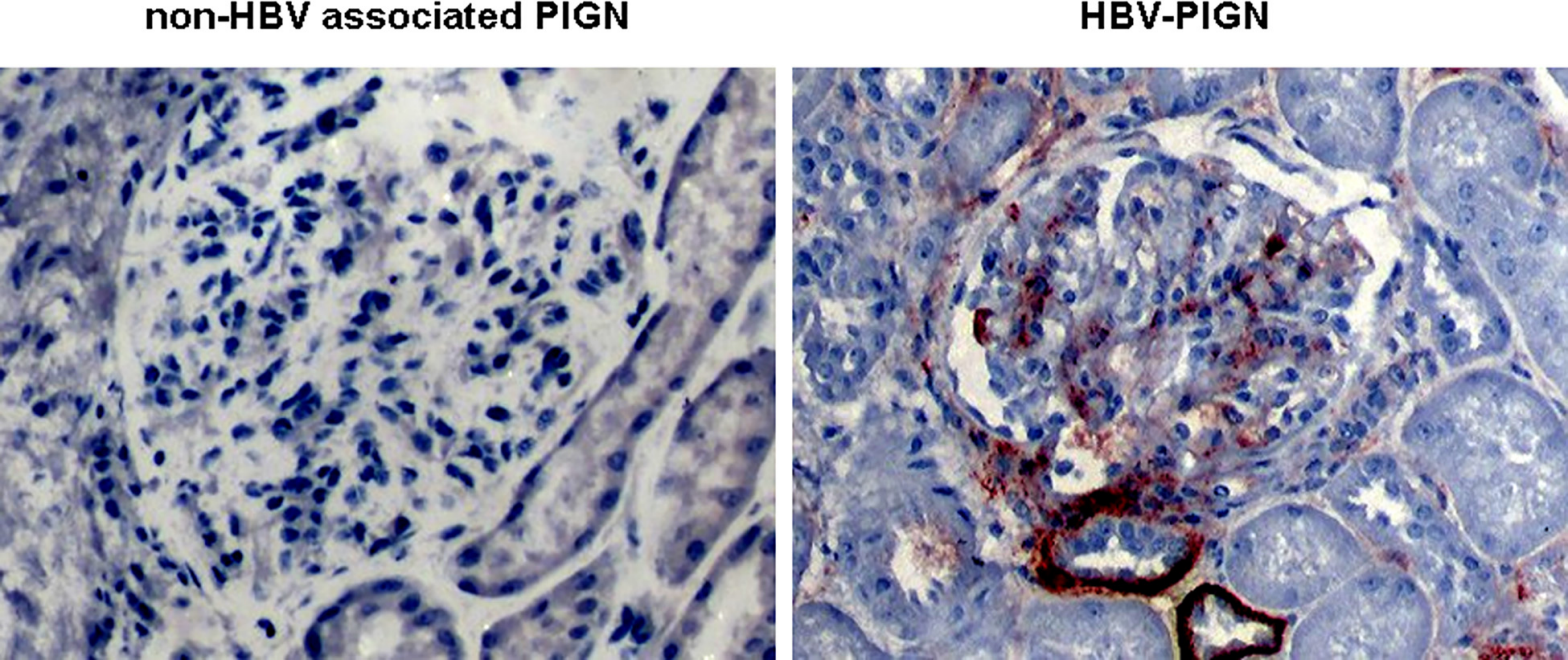
The presence of HBsAg in glomeruli of HBV-PIGN: HbsAg immunostaining was performed with a specific antibody and visualized by the immunoperoxidase method. Representative pictures show dark red positive stains in glomerulus and in the basal membrane of tubules in HBV-PIGN. The stains are negative in non-HBV associated PIGN.

HBsAg staining was positive in glomeruli in all patients with HBV-PIGN, while it was negative in patients with non-HBV associated PIGN (Fig 2). Positive HBsAg staining was also seen in areas like the juxtaglomerulus, the tubules, the interstitium, and the vasculature of patients with HBV-PIGN. Staining for immunoglobulins A, G and M, complement 1q, 3 and 4, and fibrinogen in glomeruli were all positive in 3 patients with HBV-PIGN and in 1 patient with non-HBV associated PIGN. The rest of patients with HBV-PIGN and non-HBV associated PIGN had positive glomerular staining in some of above immunoglobulins and complements, but no clear pattern could be surmised.

Since the levels of serum complement 4 were reduced in 4 patients with non-HBV associated PIGN while being normal in all patients with HBV-PIGN, the difference in glomerular complement 4 deposition was carefully examined. However, the stain results were generally weak in glomeruli in both HBV-PIGN and non-HBV associated PIGN, suggesting that glomerular complement 4 deposition may not be the cause of the difference in its serum levels.

**Fig 3:**
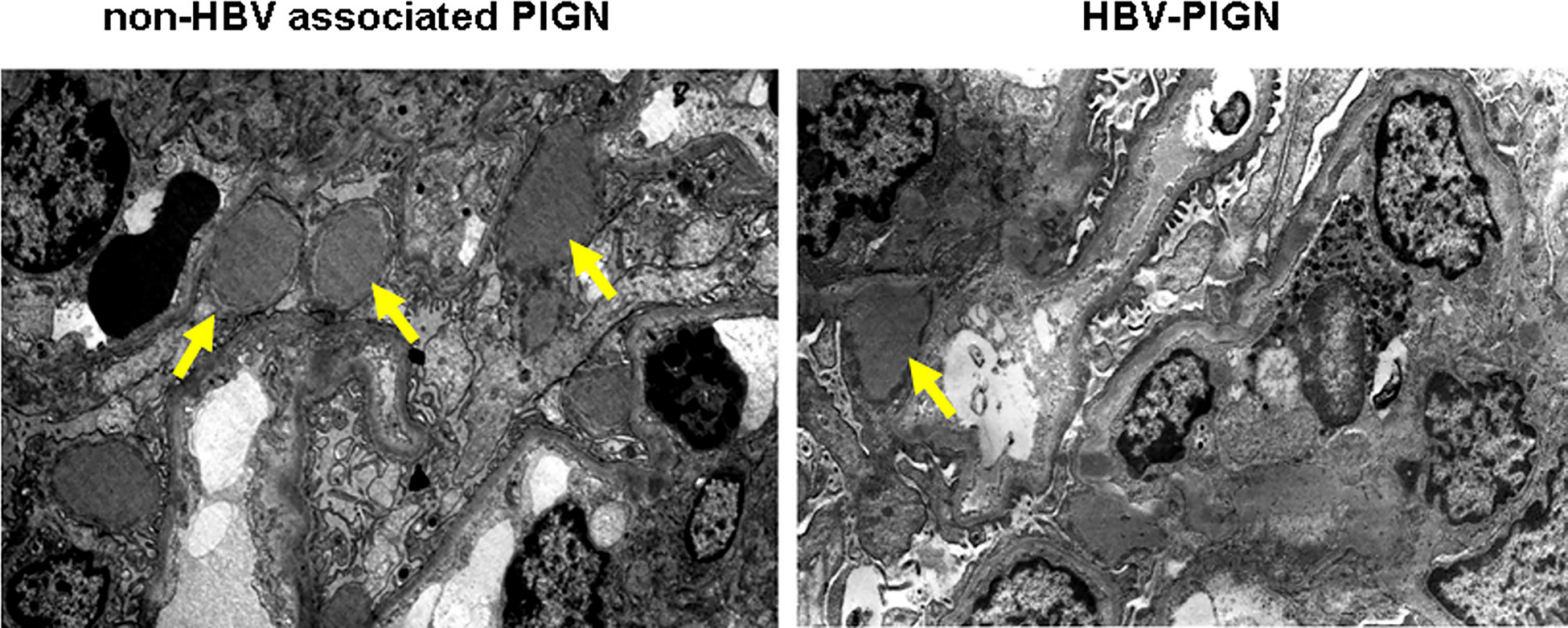
Fewer “hump-shape” sub-epithelial dense deposits in patients with HBV-PIGN. Representative electron microscopy show multiple “hump shape” sub-epithelial dense deposits (arrows) in non-HBV associated PIGN. The number of dense deposit is fewer in HBV-PIGN (arrow).

Electron microscopy examination revealed typical and multiple “hump-shape” sub-epithelial dense deposition in 7 out of 10 patients with non-HBV associated PIGN (Fig 3). In contrast, “hump-shape” sub-epithelial dense deposition was observed in only two patients with HBV-PIGN. The number of deposits was much lower in HBV-PIGN patients as compared to patients with non-HBV associated PIGN (Fig 3). Sub-endothelial and mesangium dense depositions were occasionally seen in both HBV-PIGN and non-HBV associated PIGN. No inclusion bodies or viral particles were found in HBV-PIGN. The effacement of podocyte foot processes occurred in some areas in both HBV-PIGN and non-HBV associated PIGN.

As summarized in Table 2, the renal histology of HBV-PIGN and non-HBV associated PIGN was largely indistinguishable under light microscopy. The notable differences between HBV-PIGN and non-HBV associated PIGN in pathology were the presence of HBsAg in glomeruli and fewer “hump-shape” sub-epithelial dense deposition in HBV-PIGN.

**Table 2:** Glomerular pathology.

	HBV-PIGN (n = 10)	non-HBV PIGN (n = 10)
Light microscopy		
Endocapillary proliferation	Present	Present
Neutrophils	Present	Present
Immunoglobulins	Present	Present
Complement 3 and 4	Present	Present
HBsAg (case)	10	0
HBV DNA (case)	9	0
Electron microscopy		
Sub-epithelial deposits (case)	2	7

### 3. HBV DNA

**Fig 4:**
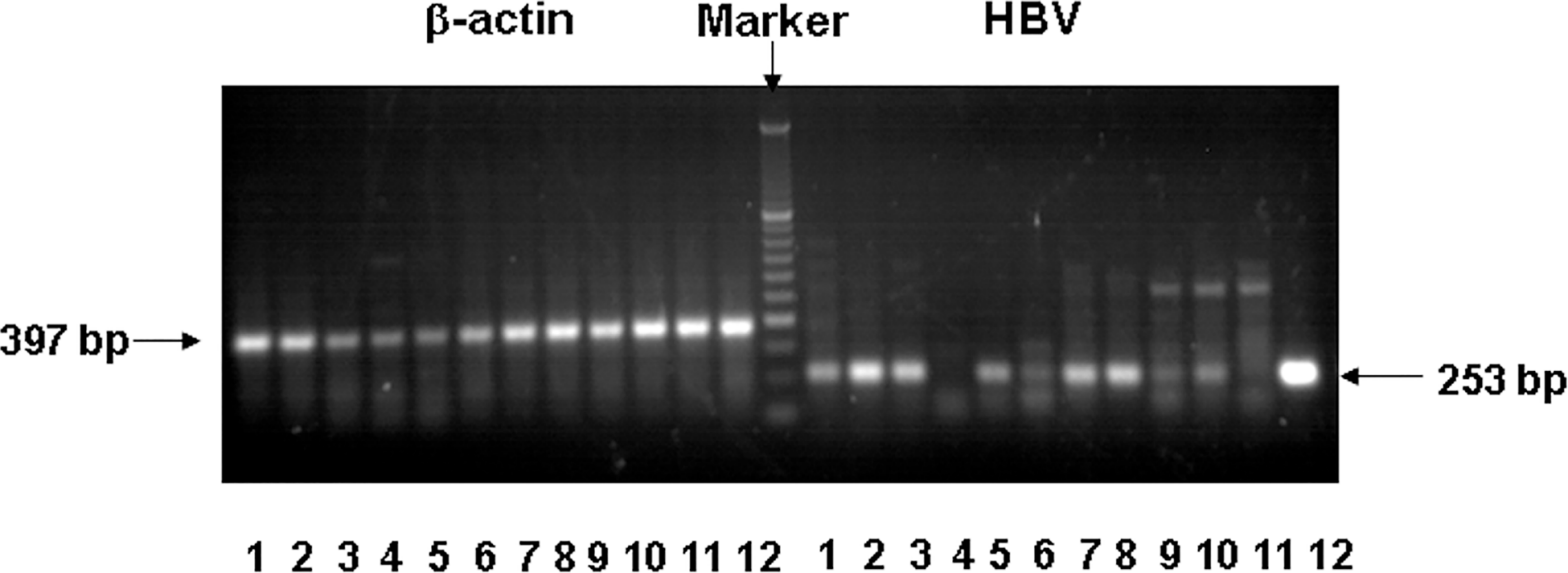
The presence of HBV DNA in glomeruli of HBV-PIGN: Glomeruli were isolated from patients with HBV-PIGN and non-HBV associated PIGN. HBV-DNA in glomeruli were detected by PCR. β-actin was used as a control to verify genomic DNA presence. PCR products with correct size were analyzed by agarose gels together with molecular markers. Representative gel shows the result of HBV-DNA and β-actin PCRs. Lane 1–10 were the glomeruli from each of 10 patients with HBV-PIGN. Lane 11 was the glomeruli from a patient with non-HBV associated PIGN. Lane 12 was a sample from HBV DNA infected liver tissue as a positive control. HBV DNA was negative in patient with non-HBV associated PIGN (lane 11) and in one HBV-PIGN patient (lane 4).

As noted previously, glomeruli were microdissected from biopsy tissues. After in-situ DNA isolation, HBV DNA was detected by PCR. HBV DNA was present in 9 out of 10 patients with HBV-PIGN (Fig 4). Sequence analysis of amplified PCR products further confirmed their origin from HBV DNA. HBV DNA was not detected in glomeruli from patients with non-HBV associated PIGN. These results are consistent with the expected post-infection status. Since there were 4 patients who were positive in both circulating and glomerular HBV DNA, we could not rule out blood origin in glomerular HBV DNA. However, 5 patients were positive only in glomerular HBV DNA, partly excluding the contribution of blood contamination.

### 4. Clinical outcomes

6 months after initial diagnosis, complete recovery, defined as the disappearance of clinical symptoms, normotensive, normal renal function, and normal urine analysis, was achieved in 2 patients with HBV-PIGN, whereas 6 patients with non-HBV associated PIGN had a complete recovery. Partial recovery, defined as the disappearance of clinical symptoms, a 50% reduction in 24 hours urinary protein excretion, and an increase in serum albumin concentration above 30 g/L, was achieved in 4 patients with HBV-PIGN and 4 patients with non-HBV associated PIGN. 4 patients with HBV-PIGN remained symptomatic and had worsening proteinuria and elevated serum creatinine 6 months after the onset of the disease, while all patients with non-HBV associated PIGN had a complete or partial recovery. Further analysis revealed that while the levels of serum albumin were below normal in patients with both HBV-PIGN and non-HBV associated PIGN at the time of diagnosis (Table 1), the levels had significantly recovered in patients with non-HBV associated PIGN, but not in patients with HBV-PIGN, after 6 months (Table 3). As such, the outcomes may be less favorable for patients with HBV-PIGN than patients with non-HBV associated PIGN.

**Table 3:** Clinical outcomes.

	HBV-PIGN (n = 10)	non-HBV PIGN (n = 10)
Completely recovery (case)	2	6
Partial recovery (case)	4	4
No improvement (case)	4	0
Serum albumin (g/L)	31.03±9.54	38.89±6.77

Patients were followed for 6 months after renal biopsy proven diagnosis. The definition of completely and partial recovery were described in the text.

## Discussion

We report here for the first time that HBV infection may also be associated with acute glomerulonephritis, observing this correlation in 10 patients with the mean age of 13 years old. This association is consistent with the high prevalence of acute post-infectious glomerulonephritis in children. We demonstrate that HBV DNA is present in the renal glomeruli of 9 out of 10 patients, and positive identification of HBsAg in the serum and glomeruli of all 10 patients. Currently, while the incidence of skin and upper respiratory streptococcus infection related acute glomerulonephritis is currently decreasing, other pathogens are now being identified that may also induce PIGN, in part due to advances in microbial screening techniques [2].

HBV infection is an endemic in China, which has a population of nearly 112 million chronic HBV carriers [16]. Associations have been observed between chronic HBV infection and chronic glomerulonephritis, and the incidence of HBV associated chronic glomerulonephritis is noticeably high in China. The most common type of HBV associated glomerulonephritis is membranous nephropathy. While a significant reduction in prevalence of HBsAg carriers and HBV associated membranous nephropathy in children after extensive HBV immunization in recent years has been reported, HBV-GN remains a significant problem [11]. Results from several clinical trials on anti-viral and immunosuppressive combined therapy suggest that such therapy may significantly ameliorate HBV-GN [12].

Renal manifestations of HBV-PIGN included nephritic syndrome, gross hematuria, nephrotic syndrome, and acute kidney injury. While similarities in clinical manifestations between HBV-PIGN and non-HBV associated PIGN were expected, there may be an increase in severity of acute glomerulonephritis in patients with HBV-PIGN due to the observed trend of increased acute kidney injury in HBV-PIGN patients. Moreover, the relatively poorer clinical outcomes in patients with HBV-PIGN after 6 months of follow-up may be related to the severity of the disease.

Pathologically, HBV-PIGN exhibited the typical changes associated with acute glomerulonephritis, including glomerular endocapillary proliferation and deposition of immunoglobulins, and deposition of complements C3 and C4. The main characteristic was the presence of HBsAg in glomerular and tubulointerstitial area in HBV-PIGN. However, the typical “hump shape” sub-epithelial dense deposition, which is commonly seen in non-HBV associated PIGN, was less frequently encountered in HBV-PIGN. The cause(s) of the difference in the amount of sub-epithelial deposition between HBV-PIGN and non-HBV associated PIGN are unknown. Since sub-epithelial dense deposition is formed as a result of immune interactions, future studies analyzing the composition of the immune complex in glomeruli of HBV-PIGN and non-HBV associated PIGN may identify the underlying cause.

Although antigen-antibody immune complex mediated inflammation in the kidneys is attributed to the pathogenesis of acute post-infectious nephritis, the exact molecular mechanisms underlying immune complex deposition and subsequent pathological actions are largely unclear. It is also unclear precisely which immune cell types are involved. Levels of serum complement 3 and 4 were reduced in patients with non-HBV associated PIGN, suggesting the involvement of activated classical and alternative complement pathways in disease process. Interestingly, 5 patients with HBV-PIGN had normal serum complement 4 levels. It is possible that there is less activation of the classical complement pathway in HBV-PIGN, since lower activation would require less complement 4 consumption. However, glomerular complement 4 staining was similarly weak in both HBV-PIGN and non-HBV associated PIGN. As such, sites other than the glomerulus and/or other mechanism may contribute to the decreased complement 4 in non-HBV associated PIGN. Nevertheless, there was noticeable difference in the amount of sub-epithelial immune complex deposition between HBV-PIGN and non-HBV associated PIGN with that contrast being potentially significant to the variance in disease severity and recovery observed. It is also noteworthy that unlike non-HBV associated PIGN, which usually occurred after acute infection, patients with HBV-PIGN did not have acute onset or acute flares of chronic hepatitis B. Instead, three out of ten patients with HBV-PIGN did have skin infection before the onset. Thus, the actual role of HBsAg and HBV DNA in glomeruli in HBV-PIGN remained to be defined.

Overall, the clinical outcome of HBV-PIGN seems to be less favorable, as indicated by no improvement in 4 patients and low serum albumin levels in this group at 6 months after onset. The cause(s) of hypoalbuminemia are not clear since only one patient with HBV-PIGN had nephrotic syndrome. It may be the result of continual presence of moderate albuminuria and decreased liver albumin synthesis. Future investigation into the nature of the roles played by HBV DNA and the complement system and other immune complexes, possibly involving increased susceptibility to acute kidney injury, may help to explain the relative poor outcome of HBV-PIGN.

In summary, we have provided evidence for an association between HBV infection and PIGN. Due to the limit in patient numbers and a relative short term of follow-up, our descriptions on clinical, laboratory and pathological pictures of HBV associated PIGN are not complete. Moreover, since we did not have a control group of PIGN who had positive serum HBV but without HBV DNA or HBsAg in glomeruli and we did not show a direct evidence of the presence of HBV DNA or HBsAg in immune complex in the glomeruli, further study is required to clearly identify HBV as a causative pathogen for PIGN.
